# The Metabolites and Mechanism Analysis of Genistin against Hyperlipidemia via the UHPLC-Q-Exactive Orbitrap Mass Spectrometer and Metabolomics

**DOI:** 10.3390/molecules28052242

**Published:** 2023-02-28

**Authors:** Zhe Li, Weichao Dong, Yanan Li, Xin Liu, Hong Wang, Long Dai, Jiayu Zhang, Shaoping Wang

**Affiliations:** 1 College of Pharmacy, Binzhou Medical University, Yantai 264003, China; 2 College of Pharmacy, Shandong University of Traditional Chinese Medicine, Jinan 250300, China

**Keywords:** genistin, hyperlipidemia, metabolites, metabolomics, UHPLC-Q-Exactive Orbitrap MS, correlation analysis

## Abstract

Genistin, an isoflavone, has been reported to have multiple activities. However, its improvement of hyperlipidemia is still unclear, and the same is true with regard to its mechanism. In this study, a high-fat diet (HFD) was used to induce a hyperlipidemic rat model. The metabolites of genistin in normal and hyperlipidemic rats were first identified to cause metabolic differences with Ultra-High-Performance Liquid Chromatography Quadrupole Exactive Orbitrap Mass Spectrometry (UHPLC-Q-Exactive Orbitrap MS). The relevant factors were determined via ELISA, and the pathological changes of liver tissue were examined via H&E staining and Oil red O staining, which evaluated the functions of genistin. The related mechanism was elucidated through metabolomics and Spearman correlation analysis. The results showed that 13 metabolites of genistin were identified in plasma from normal and hyperlipidemic rats. Of those metabolites, seven were found in normal rat, and three existed in two models, with those metabolites being involved in the reactions of decarbonylation, arabinosylation, hydroxylation, and methylation. Three metabolites, including the product of dehydroxymethylation, decarbonylation, and carbonyl hydrogenation, were identified in hyperlipidemic rats for the first time. Accordingly, the pharmacodynamic results first revealed that genistin could significantly reduce the level of lipid factors (*p* < 0.05), inhibited lipid accumulation in the liver, and reversed the liver function abnormalities caused by lipid peroxidation. For metabolomics results, HFD could significantly alter the levels of 15 endogenous metabolites, and genistin could reverse them. Creatine might be a beneficial biomarker for the activity of genistin against hyperlipidemia, as revealed via multivariate correlation analysis. These results, which have not been reported in the previous literature, may provide the foundation for genistin as a new lipid-lowering agent.

## 1. Introduction

Genistin (4′,5,7-Trihydroxyiso-flavone 7-glucoside) is one of the isoflavones found in soybeans, membranous milkvetch root, and botanical herbs from East Asia, Southeast Asia, and some Pacific islands [1]. In recent years, genistin has been repeatedly reported to have anti-oxidant, anti-inflammatory, anti-bacterial, and anti-viral activities, as well as inhibiting blood lipids [2,3,4]. Studies have shown that flavonoids also have the function of reducing blood glucose, regulating glucose metabolism, blood lipids, liver enzyme activity, and blood lipids, but their function in reducing blood lipids does not seem to be so significant [5]. Glycosides are difficult to absorb into the blood circulation due to their complicated structures [6]. Some studies have shown that the concentration of the aglycone (< 0.4μm) in plasma is lower than the IC_50_ values (10–50 μM) reported for its anti-cancer effect in vitro, even after ingestion of large amounts of genistin-containing soy products (16 mg/kg) [7]. Thus, the metabolites of genistin in vivo should be associated with its activities. However, genistin metabolites in blood are rarely found and identified using unscientific instruments with reasonable analysis strategies. Recently, Ultra-High-Performance Liquid Chromatography Quadrupole Exactive Orbitrap Mass Spectrometry (UHPLC-Q-Exactive Orbitrap MS) has been used in the discovery of trace substances in vivo based on its high resolution and high throughput [8]. In addition, a scientific analysis strategy is also one of the critical factors restricting the identification of metabolites. Among them, high-resolution extracted ion chromatography (HREIC), multiple mass defect filtering (MMDF), neutral loss filtering (NLF), and diagnostic product ions (DPIs) can be applied to the detection and identification of metabolites [9,10,11]. 

Hyperlipidemia, as a common metabolic disease, is accompanied by an increase in lipids in vivo, including total triglycerides (TG), low-density lipoprotein cholesterol (LDL-C), and total cholesterol (TC) levels and an decrease in high-density lipoprotein cholesterol (HDL-C) [12,13]. Excessive lipids are easily accumulated in the liver and lead to lipid peroxidation with the participation of reactive oxygen species (ROS). This will lead to a decrease in superoxide dismutase (SOD) with an increase in malondialdehyde (MDA) [14]. Lipid peroxidation is associated with liver damage via elevated alanine transaminase (ALT) [15]. Isoflavones seem to have better effects in treating hyperlipidemia. Puerarin, sophoricoside, and others have been reported to reduce blood lipids by regulating lipid metabolism and lipid homeostasis [16,17]. Genistin may also have a therapeutic effect on hyperlipidemia. However, the application of genistin in the treatment of hyperlipidemia has been rarely reported, and the same is true for its relevant mechanism.

Metabolomics based on UHPLC-Q-Exactive Orbitrap MS has become a useful method to reveal the mechanism of natural products or agents. Meanwhile, the metabolic differences of natural products or agents in normal and pathological conditions are related to their activities. For genistin, the beneficial function and mechanism for treating hyperlipidemia still remains mysterious, and metabolic behaviors in normal and hyperlipidemia states are fuzzy. Hence, in this study, a high-fat diet (HFD) was used to induce a hyperlipidemic rat model. The metabolic pathways of genistin in normal and hyperlipidemic rats were deeply explored via UHPLC-Q-Exactive Orbitrap MS. The effect of genistin against hyperlipidemia was determined; its mechanism was analyzed via metabolomics results and cytokines with Spearman correlation analysis. The expected results, which were never reported in the previous literature, may reveal the beneficial effect of genistein against hyperlipidemia and may also inevitably elucidate the metabolic difference between genistein in normal rats and that in hyperlipidemic rats. 

## 2. Results

### 2.1. The Establishment of Analytical Strategy

In this study, we established a comprehensive and effective strategy to discover and identify genistin metabolites by using UHPLC-Q-Exactive Orbitrap MS. Firstly, a high-quality full scan was performed with a resolution of 70,000 FWHM. Secondly, high-resolution extracted-ion chromatography was applied to withdraw the candidate data from positive and negative ion modes. Then, the candidate ions were systematically mined based on the common biological reactions and the reported metabolites in the literature. Those screened ions which we considered useful were added into the parent ion list (PIL) to obtain more accurate MS^2^ information for structure identification. Finally, the exact structures of these metabolites were resolved based on the exact molecular weight, fragmentation mode, DPIs, and information in the literature. 

Furthermore, the MMDF metabolic templates were of significance in identifying those metabolites present in low levels. In this study, four templates were set in parallel to encircle the metabolites: (1) genistin (*m/z* 431) and its conjugation templates (*m/z* 269 for deglycosylation, *m/z* 461 for hydroxylation and methylation, *m/z* 429 for glucuronidation); (2) genistein (*m/z* 271) and its conjugation templates (*m/z* 243 for decarbonylation, *m/z* 253 for dehydration, *m/z* 401 for arabino glycosylation); (3) daidzin (*m/z* 415) and its conjugation templates (*m/z* 433 for hydration, *m/z* 445 for hydroxylation and methylation); (4) daidzein (*m/z* 253) and its conjugation templates (*m/z* 223 for hydroxymethyl loss, *m/z* 257 for carbonyl hydrogenation reaction, *m/z* 225 for decarbonylation). In addition, some metabolites were also set as new templates when these metabolites were found during the subsequent identification or when the current templates did not cover the metabolic profiles of genistin. 

**Figure 1:**
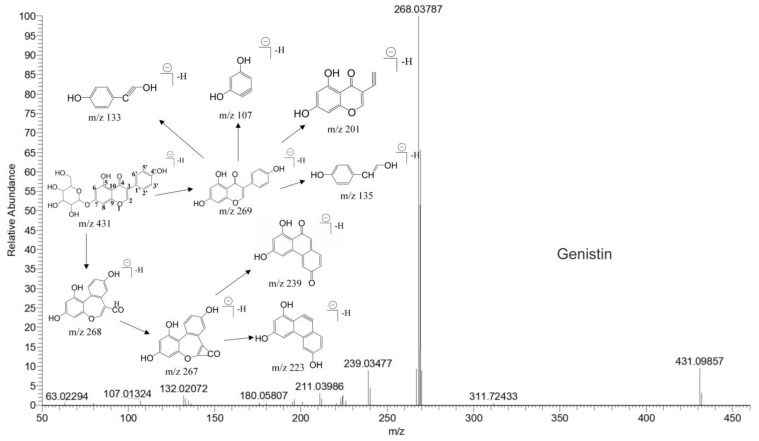
The metabolic profile of genistin.

The mass spectrum information for four prototype drugs (genistin, genistein, daidzin and daidzein) were collected and resolved via the established analysis strategy. The metabolic profile of genistin is shown in Figure 1, and other compounds are shown in the Supplementary Figures S1–S3.

### 2.2. The Identification Results of Genistin Metabolites in Normal and Hyperlipidemic Rats

The metabolites were screened and identified in plasma via UHPLC-Q-Exactive Orbitrap MS. Thirteen metabolites were detected in both positive and negative ion modes (Table 1). Among them, five metabolites were found in a positive ion mode, and eight metabolites were identified in a negative ion mode. Among them, the products of deglycosylation, decarbonylation, hydrogenation, and carbonyl hydrogenation were identified in the positive ion mode. The products of arabinosylation, glucuronidation, hydration, hydroxylation and methylation, decarbonylation, and dehydroxymethylation were identified in a negative ion mode.

#### 2.2.1. The Identification of Genistin Metabolites in Normal Rats 

Daidzein **(M0)** was eluted at 6.97 min with [M+H]^+^ ion at m/z 255.06518; its formula was calculated as C_15_H_11_O_4_. In its MS/MS spectrum, the DPIs at m/z 137 ([M+H-C_8_H_6_O]^+^) and *m*/*z* 119 ([M+H-C_7_H_4_O_3_]^+^) were generated due to RDA fragmentation [18]. 

Genistin **(M2)** exhibited the theoretical [M-H]^−^ ion at *m/z* 431.09837 (C_21_H_20_O_10_, −0.023 ppm) with a retention time of 1.24 min. Meanwhile, the fragment ion at *m/z* 92 ([M-H-C_15_H_15_O_9_]^−^) indicated the loss of B-ring, and the DPI at *m/z* 162 ([M-H-C_11_H_12_O_7_]^−^) indicated that the reaction of RDA cleavage occurred at positions 1 and 4 of **M2**. The two fragment ions were found in genistin in the MS/MS spectrum [19]. 

The glucuronidation product of daidzein **(M3)** was eluted at 0.98 min with the theoretical [M-H]^−^ ion at *m/z* 429.08273 (C_21_H_17_O_10_, 4.452 ppm). It was 176 Da higher than that of daidzein. In the MS^2^ spectra, the DPIs at *m/z* 162 ([M-H-C_12_H_14_O_7_]^−^) and *m/z* 160 ([M-H-C_12_H_16_O_7_]^−^) indicated the occurrence of an RDA reaction. The characteristic fragment ion at *m/z* 92 ([M-H-C_15_H_13_O_9_]^−^) indicated that the glucose group should be not introduced into the B-ring. The fragment ion at *m/z* 187 ([M-H-C_10_H_10_O_7_]^−^) was produced by the rupture of the A-ring [20]. 

The hydration product of daidzin **(M4)** possessed the theoretical ion [M-H]^−^ at *m/z* 433.11405 (C_21_H_21_O_10_, −0.716 ppm) with a retention time of 0.91 min. It was 18 Da higher than that of daidzin. In the MS^2^ spectra, the DPIs at *m/z* 92 ([M-H-C_15_H_17_O_9_]^−^) and *m/z* 197 ([M-H-C_6_H_10_O_5_-C_2_H_2_O_3_]^−^) indicated that the hydration reaction occurred in the C-ring [21].

The hydroxylation and methylation product of daidzin **(M5)** was 30 Da higher than that of daidzin with a retention time of 9.57 min. It exhibited the theoretical ion [M-H]^−^ at *m/z* 445.11395 (C_22_H_21_O_10_, 1.573 ppm). In the MS^2^ spectrum, the methoxy group might be added to the A-ring due to the DPI at *m/z* 407 ([M-H-C_3_H_2_]^−^), which was produced by the loss of the B-ring. The fragment ion at *m/z* 148 ([M-H-C_11_H_16_O_7_]^−^) also confirmed the above conclusion. 

The decarbonylation product of equol **(M7)** was eluted at 12.53 min and produced the theoretical [M+H]^+^ ion at *m/z* 215.10667 (C_14_H_15_O_2_, 2.714 ppm) in the positive ion mode. It was 28 Da lower than that of equol. In its MS^2^ spectrum, the fragment ion at *m/z* 149 ([M+H-C_5_H_6_]^+^) was produced due to the removal of the B-ring, and the fragment ion at *m/z* 146 ([M+H-C_4_H_5_O]^+^) was generated by the loss of positions 5 and 8 on the A-ring. 

The hydrogenation product of equol **(M8)** was eluted at 9.04 min and possessed the theoretical [M+H]^+^ ion at *m/z* 245.08077 (C_14_H_13_O_4_, 0.100 ppm). It was 2 Da higher than that of equol. In its MS^2^ spectrum, the simultaneous removal of the A-ring and the B-ring produced the DPI at *m/z* 83 ([M+H-C_10_H_10_O_2_]^+^). The fragment ions at *m/z* 93 ([M+H-C_8_H_7_O_3_]^+^), *m/z* 226 ([M+H-H_2_O]^+^), and *m/z* 69 ([M+H-C_10_H_8_O_3_]^+^) indicated that the possible reaction could only occur at **position** 3 of the C-ring. 

#### 2.2.2. The Identification of Genistin Metabolites in Hyperlipidemic Rats 

The dehydroxymethylation product of daidzein **(M10)** was eluted at 9.97 min, at which point it produced the theoretical [M-H]^−^ ion at *m/z* 223.04003 (C_14_H_7_O_3_, −1.02 ppm). In the MS^2^ spectrum, the characteristic fragment ion at *m/z* 111 ([M-H-C_5_H_4_O_3_]^−^) was produced via RDA rearrangement, which occurred on the 3’ and 6’ positions of the B-ring. The DPIs at *m/z* 183 ([M-H-C_2_O]^−^) and *m/z* 92 ([M-H-C_8_H_3_O_2_]^−^) were observed, suggesting that the hydroxyl and methyl groups were lost in the A-ring. The cleavage behavior was similar to that of the standard. 

The carbonyl hydrogenation product of daidzein **(M11)**, which was eluted at 9.37 min, contributed the theoretical [M+H]^+^ ion at *m/z* 257.08088 (C_15_H_13_O_4_, 1.691 ppm). It was 2 Da higher than that of daidzein. The DPIs at *m/z* 69 ([M+H-C_11_H_8_O_3_]^+^) and *m/z* 189 ([M+H-C_4_H_4_O]^+^) were produced due to the RDA reaction on the B-ring in its MS^2^ spectrum.

The decarbonylation product of daidzein **(M12)** exhibited the theoretical [M-H]^−^ ion at *m/z* 225.05573 (C_14_H_9_O_3_, −0.566 ppm), which was 28 Da lower than that of daidzein. In its MS^2^ spectrum, the fragment ion at *m/z* 183 ([M-H-C_2_H_2_O]^−^) was produced by the B-ring cleavage, and the fragment ion at *m/z* 165 ([M-H-C_2_H_2_O-H_2_O]^−^) confirmed our conclusion.

#### 2.2.3. Identification of Genistin Metabolites in Hyperlipidemia and Normal Rats 

The arabinylation product of genistein **(M1)** was eluted at 9.39 min and produced the theoretical [M-H]^−^ ion at *m/z* 401.08777 (C_20_H_17_O_9_, 1.134 ppm), which was 132 Da higher than that of genistein. In the MS^2^ spectra, the DPIs at *m/z* 225 ([M-H-C_5_H_8_O_4_-CO_2_]^−^), *m/z* 357 ([M-H-CO_2_]^−^), and *m/z* 313 ([M-H-CO_2_-CO_2_]^−^) confirmed the theory above. 

The decarbonylation product of genistein **(M6)**, the retention time of which was 9.87 min, was 28 Da lower than that of genistein in the positive ion mode. In the MS^2^ spectra, the DPIs at *m/z* 107 ([M+H-C_7_H_4_O_3_]^+^), *m/z* 226 ([M+H-O]^+^), *m/z* 69 ([M+H-C_10_H_6_O_3_]^+^), and *m/z* 93 ([M+H-C_8_H_6_O_3_]^+^) suggested that a decarbonylation reaction should not occur on the B-ring. However, the fragment ion at *m/z* 121 ([M+H-C_7_H_6_O_2_]^+^) indicated that the decarbonylation reaction could occur on the C-ring. 

The hydroxylation and methylation product of genistin **(M9)** was eluted at 10.09 min; it possessed the theoretical [M-H]^−^ ion at *m/z* 461.10887 (C_22_H_21_O_11_, 2.289 ppm). It was 30 Da higher than that of genistin. In the MS^2^ spectrum, the characteristic fragment ion at *m/z* 137 ([M-H-C_15_H_16_O_8_]^−^) was produced due to the RDA reaction on the C-ring. Meanwhile, the DPIs at *m/z* 407 ([M-H-C_3_H_2_O]^−^), *m/z* 92 ([M-H-C_6_H_10_O_5_-C_10_H_7_O_5_]^−^), *m/z* 425 ([M-H-2H_2_O]^−^), and *m/z* 84 ([M-H-C_6_H_10_O_5_-C_12_H_7_O_4_]^-^) indicated the methoxyl group should occurr on the A-ring.

### 2.3. Proposed Metabolic Pathways of Genistin

**Figure 2:**
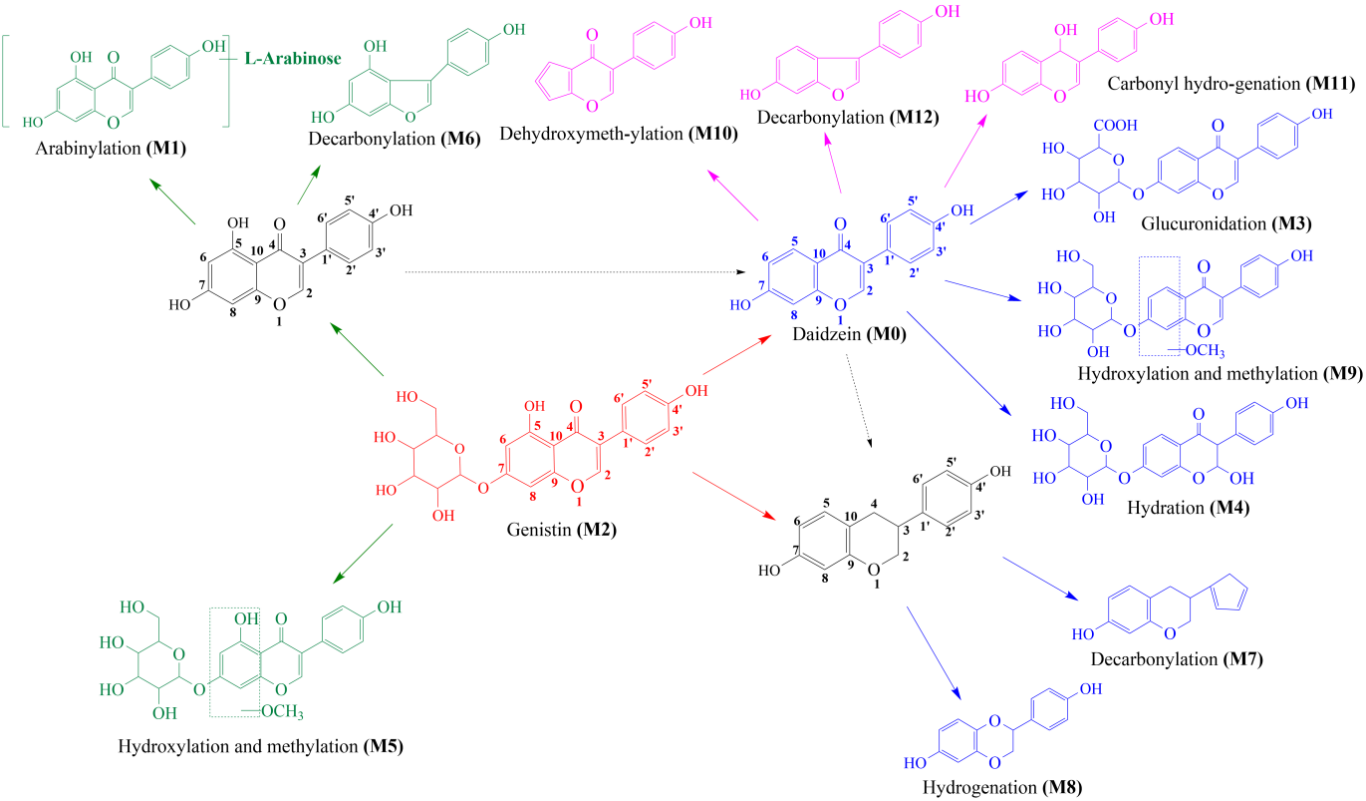
The proposed metabolic patterns of genistin in normal and hyperlipidemic rats. The parent nucleus of genistin was labeled with red; the common metabolites in hyperlipidemic and normal administration rats were labeled with green; metabolites in normal administration rats were labeled with blue; and metabolites in hyperlipidemic administration rats were labeled with pink.

Thirteen metabolites (parent drug included) were found in normal and hyperlipidemic rats after oral administration of genistin. The proposed metabolic pathways of genistin are illustrated in Figure 2. Genistin **(M2)** served as a metabolic center to gradually produce secondary metabolites. For the common metabolic pathways of normal and hyperlipidemic rats, genistin was metabolized to **M6**, **M1**, and **M9** mainly by decarbonylation, arabinylation, hydroxylation, and methylation. The reaction of three metabolites should be the conventional pathway of genistin metabolism in vivo. In normal rats, genistin was metabolized to daidzein **(M0)**, and **M0** was further metabolized to M3, **M5**, **M4**, **M7**, and **M8** underwent glucuronidation, hydroxylation and methylation, hydration, decarbonylation, and hydrogenation, respectively. These metabolic reactions might be stress patterns of genistin being cleared in a normal organism. In hyperlipidemic rats, genistin was metabolized mainly to **M10**, **M12**, and **M11** by dehydroxymethylation, decarbonylation, and carbonyl hydrogenation. It has been suggested that genistin may be metabolized into these three metabolites which are involved in the pathogenesis of hyperlipidemia.

### 2.4. Genistin Reduced Lipid Factor Levels and Hepatic Lipid Accumulation in Rats with Hyperlipidemia Induced by HFD

**Figure 3:**
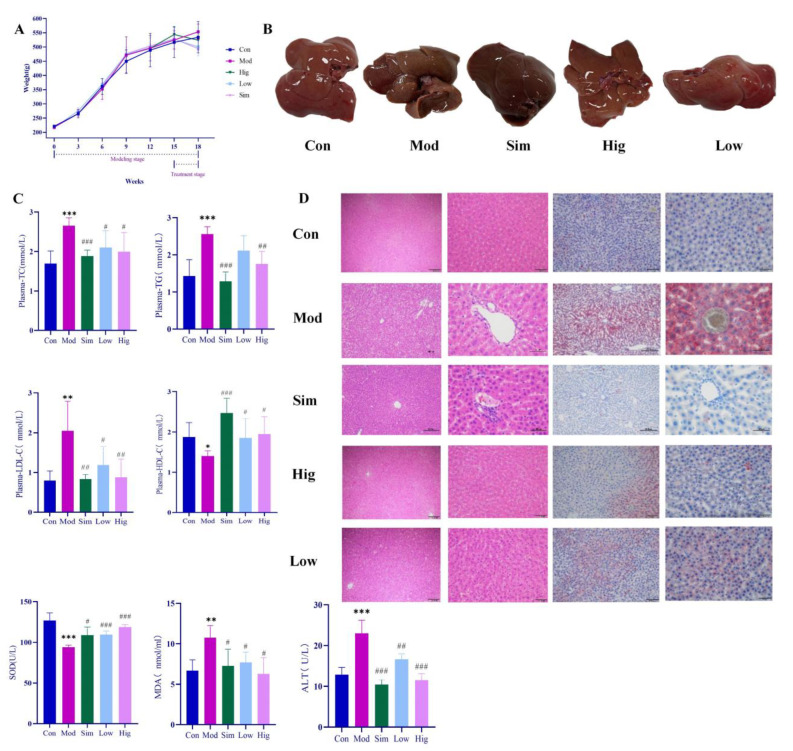
Results of anti-hyperlipidemia activity of genistin. Con: control group; Mod: high-fat diet group; Hig: genistin high dose treatment group; Low: genistin low dose treatment group; and Sim: simvastatin calcium administration group. (**A**) Statistics of body weight change in rats in test groups. (**B**) Rat liver photographs in test groups. (**C**) Hypolipidemic effect of genistin (TC, TG, LDL-C, HDL-C, ALT, SOD, and MDA levels in the plasma of rats). (**D**) The results of H&E staining (**left**, 100 μm and 400 μm) and Oil Red O staining (**right**, 200 μm and 400 μm). n = 6. * *p* < 0.05, ** *p* < 0.01, and *** *p* < 0.001, Con vs. Mod; and ^#^ *p* < 0.05, ^##^ *p* < 0.01, and ^###^ *p* < 0.00, Sim vs. Mod, Hig vs. Mod, Low vs. Mod.

During the experiment, the body weights of rats in all groups were measured per week. As shown in Figure 3, HFD could rapidly increase the body weight of rats. However, when we gave genistin, it significantly slowed down the weight gain, and the effect was consistent with simvastatin. At the end of the experiment (18 weeks), we found that the levels of plasma TC, TG, LDL-C in Mod rats were significantly increased (*p* < 0.01), whereas HDL-C was decreased compared with that in rats in Con (*p* < 0.01), which indicated that HFD could indeed cause hyperlipidemia in rats. After treatment, simvastatin and genistin at different doses significantly reduced the levels of plasma TC, TG, and LDL-C (*p* < 0.01), and elevated the standard of HDL-C (*p* < 0.01). This beneficial function was also observed in the liver color of rats in all groups. All results indicated that genistin has the remarkable function of regulating blood lipids.

For Oil red O staining, more lipids accumulated in the liver after being raised with HFD. As we know, the excessive lipids could cause a lipid peroxidation reaction with the participation of reactive oxygen species, and lipid peroxidation was associated with cell permeability, DNA damage, and protein synthesis disorders. In Mod rats, HFD significantly increased the content of MDA (*p* < 0.05) while decreasing the level of SOD compared with that in Con rats (*p* < 0.05). The result indicated that excessive HFD could accelerate the process of lipid peroxidation in vivo. Moreover, lipid peroxidation emerged as having a negative relationship with liver function. For H&E staining, the livers of rats in Mod indeed showed increased symptoms of cavitation and fibrosis, which also corroborated the above statement. Moreover, these elevated levels of ALT in Mod rats also corroborated the abnormal liver function compared with Con (*p* < 0.05). Surprisingly, genistin obviously reversed the above situation; this means that genistin could reduce lipid accumulation in the liver. Concurrently, genistin also significantly reduced the level of MDA (*p* < 0.05) and increased the content of SOD (*p* < 0.05). It was shown that genistin has an inhibitory effect on lipid peroxidation. Genistin also had a beneficial effect on liver function by reducing fibrosis and vacuolation of liver cells. This result was also reflected in the decreased ALT due to genistin. In brief, genistin could reduce HFD-induced lipid factor levels and hepatic lipid accumulation in rats.

### 2.5. The Mechanism Analysis of Genistin against Hyperlipidemia via Plasma Metabolomics

**Figure 4:**
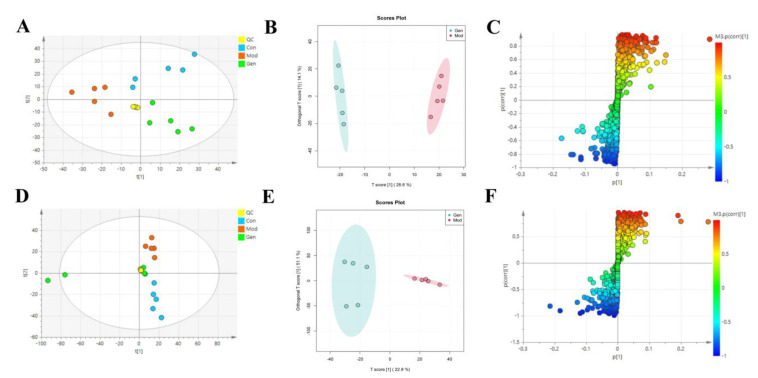

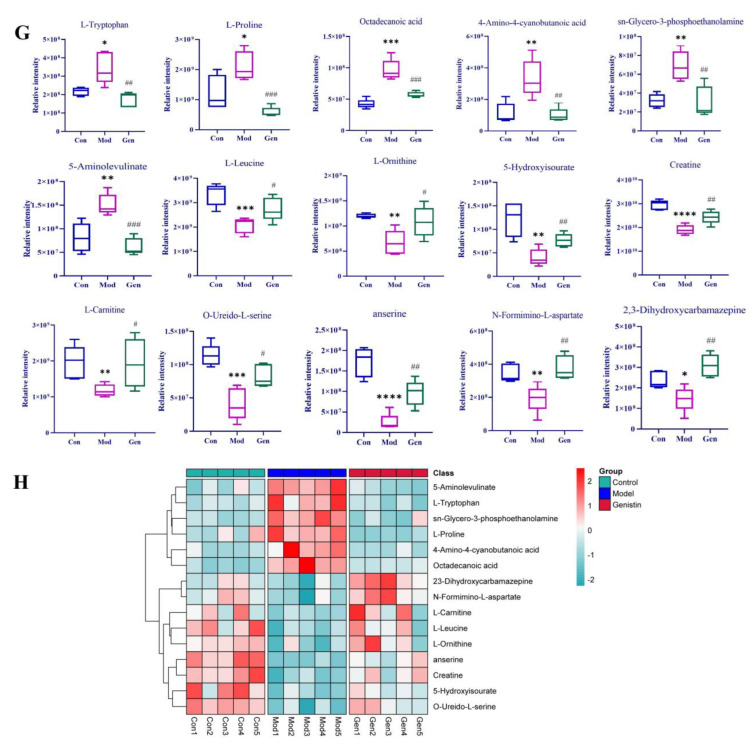
The effects of genistin on metabolic parameters in hyperlipidemic rats and correlation analysis. (**A**) Negative PCA score plot. (**B**) Negative OPLS-DA plots (R2X = 0.424, R2Y = 0.989, Q2 = 0.869). (**C**) Negative S-plots. (**D**) Positive PCA score plot. (**E**) Positive OPLS-DA plots (R2X = 0.458, R2Y = 0.984, Q2 = 0.875). (**F**) Positive S-plots. (**G**) Results of 15 differential metabolites vs. Gen, Con, and Mod. (**H**) Heat map of the differential metabolites in the plasma of rats (Red and blue represent increased and decreased metabolite content, respectively). Gen: genistin high dose treatment group. * *p* < 0.05, ** *p* < 0.01, *** *p* < 0.001, and **** *p* < 0.0001, Con vs. Mod; ^#^ *p* < 0.05, ^##^ *p* < 0.01, and ^###^ *p* < 0.001, Gen vs. Mod.

To determine the underlying mechanism of genistin in the treatment of hyperlipidemia, multivariant PCA and OPLS-DA were applied with SIMCA-P 14.0 Demo software (Figure 4A,B,D,E). PCA is an unsupervised cluster analysis model without preprocessed datasets. It can be used to evaluate differences in metabolic profiles of Con, Mod, and Gen.

Above all, the QC sample could be gathered together in PCA score plots of positive and negative ion modes, illustrating the stability of the analysis system during data collection. Furthermore, the separation trend between Con and Mod were highly obvious. This consequence was reflected in the destructive effect of HFD on metabolic profiling in rats. As expected, Mod was also significantly different from Gen, the metabolic profiling of which was more inclined to that of the Con. Next, PLS-DA was adopted to verify the rationality of the data model. This supervisory analysis model revealed the difference between Mod, Con, and Gen. This simulation coefficient of R2Y (0.989, 0.984) and Q2 (0.869, 0.875) in positive and negative ion modes revealed the reliability of the model, and the 200 permutation tests also confirmed the above results.

OPLS-DA was utilized to identify significantly changed metabolites after genistin treatment. On the score plots of OPLS-DA in positive and negative ion modes, there were obvious separation trends between Con and Mod and between Mod and Gen. Subsequently, the S-plot with the threshold of VIP > 1.0 and *p* < 0.05 could be used to screen and identify differential metabolites in the OPLS-DA model (Figure 4C,F). Finally, a total of 15 endogenous metabolites were significantly altered between the Con and Mod (Figure 4G, Table 2). Among them, six metabolites were obviously up-regulated (*p* < 0.05), and nine metabolites were significantly decreased (*p* < 0.05) with HFD intervention. Nevertheless, genistin could still obviously down-regulate six metabolites (*p* < 0.05), including L-Tryptophan, L-Proline, Octadecanoic acid, 4-Amino-4-cyanobutanoic acid, sn-Glycero-3-phosphoethanolamine, and 5-Aminolevulinate. Genistin also up-regulated 9 of 15 metabolites, involving L-Leucine, Creatine, L-Carnitine, O-Ureido-L-serine, anserine, N-Formimino-L-aspartate, L-Ornithine, 2,3-Dihydroxycarbamazepine, and 5-Hydroxyisourate. Approximate changes in multiples of metabolites are shown in Additional Table 1. These results demonstrated that genistin indeed reverses metabolic disorders caused by HFD. The cluster analysis with heat map was used to verify the above hypothesis (Figure 4H). We found that the metabolic profiling of genistin in the treatment of hyperlipidemia was remarkably similar to that of the Con, whereas the contrary was true for that of the Mod. The results showed that the levels of metabolites in the plasma were significantly different between the Con and Mod rats, and that the levels in the Gen tended to recover to those of the Con, suggesting that genistin could correct the abnormal levels of plasma metabolites in hyperlipidemic rats.

### 2.6. The Enrichment Analysis of Metabolic Pathways

**Figure 5:**
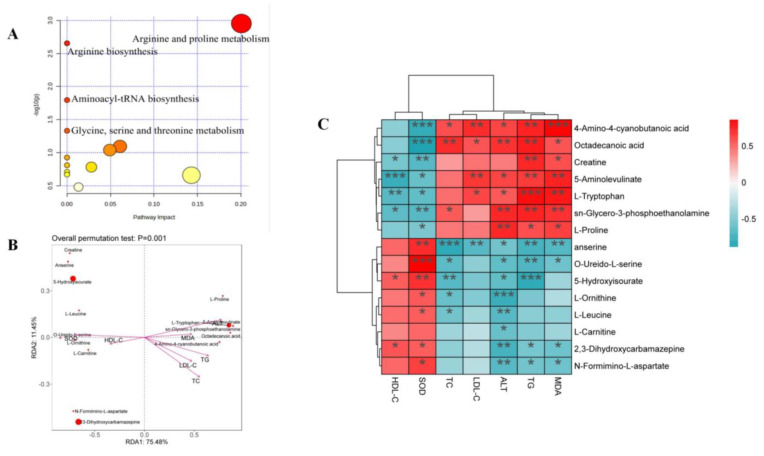
(**A**) Plasma sample pathway analysis between Con vs. Mod vs. Gen. The ordinate was the *p*-value of pathway enrichment analysis, and the abscissa was the pathway impact values from the pathway topology analysis. The larger the value, the stronger the correlation. (**B**) Results of db-RDA analysis. (**C**) Heatmap of the correlations between plasma lipid parameters and plasma metabolites. * *p* < 0.05, ** *p* < 0.01, and *** *p* < 0.001.

Fifteen differential metabolites were imported to the MetaboAnalyst 5.0 dataset, and their results revealed the metabolic pathway of genistin against hyperlipidemia (Figure 5A). The results showed that these metabolites in the plasma were responsible mainly for arginine and proline metabolism and arginine biosynthesis, and so on. Therefore, these metabolic pathways should be classified as target pathways, which were associated with the beneficial activity of genistin.

Moreover, the Spearman correlation analysis was used to determine the significant correlations between metabolites and cytokines. As shown in Figure 5C, six metabolites, including 4-Amino-4-cyanobutanoic acid, octadecanoic acid, L-Proline, L-Tryptophan, sn-Glycero-3-phosphoethanolamine, and 5-Aminolevulinate, showed positive correlation with TC, TG, LDL-C, ALT, and MDA and were negatively related to SOD and HDL-C. Interestingly, six metabolites seemed to be selectively related to cytokines. Among them, 4-Amino-4-cyanobutanoic acid and octadecanoic acid indeed showed positive correlation with TG, TC, and LDL-C (*p* < 0.05), illustrating that two metabolites might aggravate the damage of hyperlipidemia to the body. The insignificant relationship between two metabolites and HDL-C also proved that they focused only on the transport of total cholesterol from the liver to the plasma. In addition, 4-Amino-4-cyanobutanoic acid and octadecanoic acid were all negative correlated with SOD (*p* < 0.001); the result suggested that they were associated with lipid peroxidation in the liver. 5-Aminolevulinate, sn-Glycero-3-phosphoethanolamine, and L-Tryptophan all showed negative correlations with SOD and HDL-C (*p* < 0.05). 5-Aminolevulinate, sn-Glycero-3-phosphoethanolamine, and L-Tryptophan could not only induce hyperlipidemia but also cause adverse effects on liver function based on the positive relationship between these compounds and TG, MDA, ALT, and LDL-C (*p* < 0.05).

The relationships between nine other metabolites and cytokines were contrary to the aforementioned situation. For indicators, all metabolites were negatively correlated with ALT (*p* < 0.05), illustrating the function of liver protection. However, three of nine metabolites, namely anserine, creatine, and O-Ureido-L-serine, were negatively correlated with TG, TC, and LDL-C (*p* < 0.05). 

Among those metabolites, only creatine could show the negative correlation with the above three factors (*p* < 0.01) and was positively correlated with HDL-C (*p* < 0.05). The result suggested that creatine seemed to show a unique function in alleviating hyperlipidemia. Moreover, the correlations between creatine and SOD and MDA also confirmed that creatine could inhibit lipid peroxidation in the liver. Other metabolites that could demonstrate this function were anserine, 2,3-Dihydroxycarbamazepine, and N-Formimino-L-aspartate (*p* < 0.05). 

Distance-based redundancy analysis (db-RDA) was applied to determine the differential biomarkers of genistin against hyperlipidemia. 4-Amino-4-cyanobutanoic acid and octadecanoic acid were negatively associated with SOD while showing a positive correlation with TG, TC, and LDL-C. In addition, creatine was more closely associated with cytokines than other metabolites. Thus, creatine should be considered a beneficial biomarker for genistin in the treatment of hyperlipidemia.

## 3. Discussion

The metabolic behaviors of drug or natural products in vivo have always been the focus of study for their continued development. The reactions involved in their metabolic pathways may be associated with the involved targets or endogenous pathways. In addition, the metabolic differences of drugs or natural products under normal and pathological conditions have been reported in the previous literature [22,23,24], which may provide some information about their mechanism. In this study, a high dose of genistin (150 mg/kg) was given orally to normal SD rats and hyperlipidemic SD rats [25]. Thirteen metabolites in plasma were found via UHPLC-Q-Exactive Orbitrap MS with an efficient analysis strategy. Among them, three metabolites were detected in both normal and pathological rats, involving the reactions of decarbonylation **(M6)**, L-arabinylation **(M1)**, and hydroxylation and methylation **(M9)**. Meanwhile, seven metabolites were found only in rats administered normally, such as deglycosylation **(M0)**, glucuronidation **(M3)**, hydroxylation and methylation **(M5)**, hydration **(M4)**, decarbonylation **(M7)**, and hydrogenation **(M8)** products of genistin. Based on the above results, we speculated that the reactions of three metabolites detected in both normal and pathological rats should be the routine pathways for genistin metabolism in vivo, and metabolic reactions of seven metabolites in normal rats might be stress patterns of genistin being cleared in a normal organism. Three other metabolites could be detected only in hyperlipidemic rats, including the reactions of dehydroxymethylation **(M10)**, decarbonylation **(M12)**, and carbonyl hydrogenation **(M11)**. This fact demonstrates that genistin may be metabolized into three metabolites to participate in the pathogenesis of hyperlipidemia, or three metabolites have some advantages in the treatment of hyperlipidemia with genistin. It is worthwhile for this latent problem to be studied in the future.

Isoflavones, also known as estrogen-like substances, have been proven to have remarkable abilities in regulating hormone homeostasis, cell proliferation, and metabolic regulation in women, such as soy isoflavone and Pueraria isoflavones [26,27,28]. In the next experiment, the special function of genistin was evaluated in HFD-induced hyperlipidemic rats. At first, HFD could disturb the lipid homeostasis in rats and accelerate the process of lipid peroxidation in liver [29]. The levels of ALT corroborated the above conclusions compared with normal rats. Secondly, genistin obviously reversed the disorder in vivo caused by HFD (*p* < 0.05); this effective activity was also confirmed by pathological results. Thirdly, hyperlipidemia was characterized by abnormally elevated levels of TC, TG, and LDL-C and abnormally reduced HDL-C levels. Genistin significantly improved this pathological change. At present, the research of genistin in the treatment of hyperlipidemia has never been mentioned, such that these results can construct the foundation for genistin as a new lipid-lowering agent. 

Finally, the mechanism behind genistin in the treatment of hyperlipidemia was preliminarily elucidated using metabolomics. In PCA and OPLS-DA score plots, the metabolic profile of genistin in rats, which was similar to that of normal rats, was verifiably separated in rats with hyperlipidemia induced by HFD. The levels of 15 metabolites in Mod rats were significantly adjusted compared with Con rats (*p* < 0.05), and these metabolites were significantly reversed by genistin (*p* < 0.05), which belonged to the metabolic pathways of arginine and proline metabolism and arginine biosynthesis. Spearman correlation analysis and db-RDA revealed that creatine should be considered a beneficial biomarker of genistin against hyperlipidemia. Creatine in mammal body could be synthesized from arginine, methionine, and proline found in the kidney, liver, and pancreas [30,31,32]. As an energy source that can be endogenously synthesized or obtained through diet and supplement, creatine is involved in cell metabolism through adenosine triphosphate (ATP) supplementation, which provides energy for skeletal muscles, organs, and tissues [33]. The deposition of lipids leads to the loss of ATP in the body, and creatine could promote the process of lipid β oxidation to release more ATP in the participation of multiple targets, such as adenosine 5’-monophosphate (AMP)-activated protein kinase (AMPK), peroxisome-proliferator-activated receptors (PPARs), sterol-regulatory element-binding proteins (SREBPs) [34]. Certainly, the relationship between creatine and lipids also reduces lipid peroxidation in the body [35]. In this study, genistin could significantly promote creatine production through the arginine and proline metabolic pathways. However, the relationship behind genistin, creatine, and the metabolic pathway remains mysterious, and the role of three genistin metabolites in hyperlipidemic rats should not be ignored. 

## 4. Materials and Methods

### 4.1. Chemicals and Reagents

The reference substances of genistin, genistein, daidzin, and daidzein were commercially provided by Chengdu Must Biotechnology Co., Ltd. (Chengdu, China) with a purity ≥98% via UV-UHPLC. Their structures were fully elucidated by comparing the spectral data (ESI-MS and ^1^H, ^13^C NMR). The kits of total cholesterol (TC), total triglyceride (TG), high-density lipoprotein cholesterol (HDL-C), alanine aminotransferase (ALT), malondialdehyde (MDA), superoxide dismutase (SOD), and low-density lipoprotein cholesterol (LDL-C) were purchased from Nanjing Jiancheng Bioengineering Institute (Nanjing, China). In addition, the other reagents and solvents, which met the requirements of analytical experiments, were purchased from Beijing Chemical Works (Beijing, China). Simvastatin was supplied by Merck Pharmaceuticals Ltd (Hangzhou, China). Ultrapure water was derived from Milli-Q Gradient Å 10 water purification system (Millipore, Billerica, MA, USA). 

### 4.2. The Identification of Genistin Metabolites in Normal and Hyperlipidemic Rats

#### 4.2.1. Establishment of Animal Model

A total of 12 male Sprague–Dawley (SD) rats (SPF level, weighing 180–200 g) were purchased from Jinan Pengyue Experimental Animal Breeding Co., Ltd. (Shandong, China, SYXK (RU)2019-0003). Before the experiment, all animals had to be maintained under standard animal room conditions (temperature 24 ± 2 °C, humidity 55–60%, 12/12 h light/dark cycles) with standard feed and water ad libitum for 1.0 week. Afterward, all rats were randomly divided into the control group (6 rats) and the hyperlipidemic group (6 rats) according to body weight. The rats in the normal group were fed normal rodent chow (Pengyue, Shandong, China), and those in the hyperlipidemic group were fed with a high-fat diet, containing a standard chow diet (65%), sucrose (20%), lard (15%), cholesterol (5%), sodium cholate (5%), and 5% yolk powder (Huafukang, Beijing, China) for 15 weeks. After 15 weeks, compared with the control group, the levels of TC, TG, and LDL-C in the hyperlipidemic group were significantly increased, and the level of HDL-C was significantly decreased, indicating that the animal model was successfully established. 

#### 4.2.2. Collection and Preparation of Plasma Samples

All rats in both groups were given genistin (150 mg/kg) via oral administration. After oral administration, the blood samples (about 0.5 mL) were taken from the rats in the normal and hyperlipidemic groups at different times of 0.5, 1, 1.5, 2, 4, and 6 h. The obtained samples were placed in the anticoagulant EP tubes of heparin sodium. After resting for 10.0 min, each blood sample was centrifuged for 15.0 min (3500 rpm, 4 °C). An amount of 100.0 μL of upper plasma was taken from the rats in the normal administration group at each time point to obtain mixed plasma. Cold methanol (3.0 mL) was added to mixed plasma samples (1.0 mL) for precipitation, and the supernatant was obtained via centrifugation for 10.0 min (4000 rpm, 4 °C). Plasma of the two groups were blown dry with a nitrogen blow dryer and stored in a refrigerator at −80 °C until use. Before analysis, samples of these two groups were redissolved in 300 μL methanol and centrifuged at 20,000 rpm.

#### 4.2.3. Collection of UHPLC-Q-Exactive Orbitrap MS Data

The unsearchable metabolites were determined via UHPLC-Q-Exactive Orbitrap MS. Firstly, LC analysis was performed on a DIONEX Ultimate 300 UHPLC system (Thermo Fisher Scientific, MA, USA) with a binary pump, an autosampler, and a column oven. The chromatographic separation was performed with an ACQUITY UPLC BEH C18 column (2.1mm × 100 mm, 17 μm, Waters, Milford, MA, USA). The column temperature was maintained at 35 °C, and a 3.0 μL sample was injected at a flow rate of 0.3 mL/min. The mobile phases consisted of 0.1% formic acid aqueous solution (A) and acetonitrile (B). The elution gradient was set as follows: 0–5 min, 5–30% (B); 5–10 min, 30–50% (B); 10–27 min, 50–90% (B); 27–27.1 min, 90–5% (B); 27.1–30 min, 5% (B). 

Subsequently, the UHPLC-MS analysis was performed on a Q Exactive MS (Thermo Fisher Scientific, MA, USA). The data acquisition parameters in the positive and negative ion modes were set as follows: spray voltage of 3000 V (positive)/3500 V (negative); capillary voltage of −35 V; sheath gas flow rate of 30 arb; auxiliary gas flow rate of 10 arb; capillary temperature of 325 °C (positive)/350 °C (negative); tube lens of +110 V (positive)/−110 V (negative). All metabolites were detected using full-scan MS analysis (*m/z* 70–1050) at a resolving power of 70,000 FWHM. The resolution of dd-MS^2^ was set to 17,500 FWHM. The collision energy of collision-induced dissociation (CID) was 30 eV. The collected datasets were recorded and processed via a Thermo Xcalibur 2.1 workstation (Thermo Scientific, Bremen, Germany). 

### 4.3. Anti-Hyperlipidemia Analysis of Genistin in Rats Induced by HFD

#### 4.3.1. Sectionalization and Administration

A total of 30 male Sprague–Dawley (SD) rats (SPF level, weighing 180–200 g) were purchased from Jinan Pengyue Experimental Animal Breeding Co., Ltd. (Shandong, China, SYXK (RU)2019-0003). They were used to evaluate the anti-hyperlipidemic activity of genistin. The modeling method was based on the above method “4.2.1.”.

Then, the rats in the hyperlipidemic group were again allocated into the four following groups: the model group (Mod, n = 6); simvastatin group (Sim, n = 6) at the dose of 5 mg/kg/d (drug weight/body weight/day); genistin high-dose group (Hig, n = 6) at the dose of 5 mg/kg/d; and genistin low-dose group (Low, n = 6) at the dose of 2.5 mg/kg/d [36]. All drugs were administered orally to the respective rats. Except for the Con group, other rats were still fed a high-fat diet for the 3 weeks of treatment. 

#### 4.3.2. Collection and Preparation of Biological Samples

At the end of the experiment, all rats were fasted for 12 h with only deionized water. Then, the rats from five groups were killed in parallel using 10% chloral hydrate. All abdominal aortic blood samples were collected from each rat in each group and placed in EP tubes coated with heparin sodium and were centrifuged (3500 rpm) for 15.0 min at 4 °C, and the supernatants were taken for testing. The livers of all rats were taken out and rinsed with normal saline. Some livers were immersed in 4% paraformaldehyde for histopathological analysis. The remaining livers were rapidly quenched in liquid nitrogen and stored at −80 °C until use.

The levels of TG, TC, LDL-C, HDL-C, ALT, SOD, and MDA in plasma samples from all rats were measured using a microplate reader (SpectraMax iD5, Pleasanton, CA, USA) [37,38,39]. The hepatic tissues fixed in 4% PFA were dehydrated and embedded in paraffin, cross-sectioned into 4 µm-thick slices, and stained with hematoxylin–eosin (H&E). The sections of the remaining liver tissues were cleaned with PBS and cultured with 60% isopropanol for 5.0 min and then dyed in 0.5% Oil Red O staining liquid (Sigma, St Saint Louis, MO, USA) for 20.0 min. After being cleaned by PBS, all sections were stained with hematoxylin stain (Solarbio Science and Technology, Beijing, China) for 2.0 min [40]. The abovementioned indicators were all used to evaluate the anti-hyperlipidemia function of genistin.

### 4.4. The Mechanism Analysis of Genistin against Hyperlipidemia via Metabolomics

#### 4.4.1. Preparation of Biological Samples

In total, 200.0 μL plasma from each rat in each group was taken and added into an 800.0 μL mixture of cold methanol and acetonitrile (1:4). After 5.0 min, the miscible liquids were centrifuged at 4000 rpm for 10.0 min to obtain the supernatants [41]. All supernatants were rapidly dried with nitrogen and stored in a refrigerator at −80 °C until use. In addition, a 10 µL solution from each plasma sample was mixed and marked as quality control (QC) samples. The stability of the instrument needed to be calibrated using QC samples after every 5 plasma samples.

#### 4.4.2. Collection of UHPLC-Q-Exactive Orbitrap MS Data

LC analysis was performed on a DIONEX Ultimate 3000 UHPLC system (Thermo Fisher Scientific, MA, USA) with a binary pump, an autosampler, and a column oven. A 2.0 μL sample was injected into an Acquity UPLC BEH C18 column (100 × 2.1 mm, 1.7 μm), and the flow rate was set to 0.3 mL/min. The column temperature was 40 °C, and the mobile phases consisted of 0.1% formic acid aqueous solution (A) and acetonitrile (B). The gradient elution condition was set as follows: 0–1min, 5% (B); 1–3 min, 5–40% (B); 3–6 min, 40% (B); 6–6.5 min, 40–60% (B); 6.5–7.5 min, 60% (B); 7.5–10 min, 60–90% (B); 10–15 min, 90–5% (B); 15–20 min, 5% (B).

The UHPLC-MS analysis was performed on a Q-Exactive MS/MS (Thermo Fisher Scientific, MA, USA). An electrospray ionization (ESI) ion source was used. The samples were collected via Full MS/dd-MS^2^ scanning mode. The first-order scanning resolution was 70,000; the second-order scanning resolution was 35,000; the Fourier high-resolution scanning range was *m*/*z* 50–1050; and the ion chamber collision energy was 40%. The capillary temperature was 320 °C.The sheath gas flow rate was 30 arb; the auxiliary gas flow rate was 10 arb; and the spray voltage was 3.0 kV. 

#### 4.4.3. Multivariate Analysis of UHPLC-Q-Exactive Orbitrap MS Data

The UHPLC-Q-Exactive Orbitrap MS data were processed with Compound Discoverer 3.0 software (Thermo Fisher Scientific, Waltham, MA, USA) for noise cancellation, baseline correction, and normalization to obtain reliable datasets with some information, including *m*/*z*, peak intensities, and retention times. The relevant parameters were set to C [0–30], H [0–60], O [0–10], N [0–5], ring unsaturated double bond (RDB) [0–15], and the mass accuracy error was within 5–10^−6^.

Afterward, the processed datasets were added to the SIMCA-P 14.0 software (Umetrics, Sweden) to perform the principal component analysis (PCA) and orthogonal to partial least-squares-discriminant analysis (OPLS-DA). Among them, PCA was applied to discriminate the separation trends of all groups. OPLS-DA was used to characterize metabolic perturbation of hyperlipidemia. In addition, the S-plot scores were used to screen the differential metabolites of hyperlipidemia treated by genistin combined with other judgment methods, such as variable importance in projection (VIP) (generated in the OPLS-DA mode) and *p*-value (formed from relative intensity). Subsequently, the S-plot with the threshold of VIP > 1.0 and *p* < 0.05 could be used to screen and identify differential metabolites in the OPLS-DA model. The structures, molecular weights, and codes of differential metabolites were assigned according to the human metabolome database (What Is Dementia. Available online: http://www.alz.org/what-is-dementia.asp (accessed on 1 February 2023)). Finally, the Spearman correlation analysis and db-RDA analysis were used to determine the relationship between lipid factors and differential metabolites (https://www.bioincloud.tech/ (accessed on 1 February 2023)). 

### 4.5. Statistical analysis

The statistical analysis of the data was performed using SPSS 22.0 software (Chicago, IL, USA). An unpaired Student’s *t*-test was performed for a two-group comparison. For multiple comparisons, ANOVA was used. *p* < 0.05 was defined as statistically significant. The statistical analyses and figures were performed using GraphPad Prism 8.0 software (Santiago, MN, USA). Fasting body weight data were analyzed using one-way ANOVA. 

## 5. Conclusions

In this study, the metabolic differences and similarities of genistin in normal rats and hyperlipidemic rats were compared. These results are able to provide the foundation for the metabolic mechanism of genistin in pathological and normal rats. The efficacy results revealed the explicit function of genistin against hyperlipidemia, which has been rarely reported; thus, the results demonstrate the possibility of genistin as a new lipid-lowering agent. 

The mechanism on genistin in the treatment of hyperlipidemia was preliminarily elucidated using metabolomics. Genistin may treat hyperlipidemia by regulating the level of creatine, which can be produced by the arginine and proline metabolic pathway in vivo. However, there are some limitations of the present study. Firstly, the certain relationship between the differences in metabolic behavior and metabolic pathways of genistin in normal and hyperlipidemic rats and the results of pharmacodynamics is still unclear. Secondly, it is also ambiguous whether there are differences in the levels of this metabolite. Finally, the character of genistin metabolites in the treatment of hyperlipidemia by mediating the level of creatine in vivo should also not be ignored. In summary, the relationship between genistin and hyperlipidemia may be further revealed in subsequent studies. 

## Figures and Tables

**Table 1:** Summary of the detection of genistin metabolites in plasma of normal rats and hyperlipidemic rats via UHPLC-Q-Exactive Orbitrap MS method.

Peaks	Product	Ion Mode	Rt/min	Formula	Theoretical Mass (*m/z*)	Experimental Mass (*m/z*)	Error/ ×10^−6^	MS/MS Fragment Ions	Identification	Con	Mod
M0	daidzein	P	6.97	C_15_H_11_O_4_	255.06518	255.06543	0.959	MS^2^[255]: **255(100)**, **137(16)**, 227(9), 237(4), **119(1)**	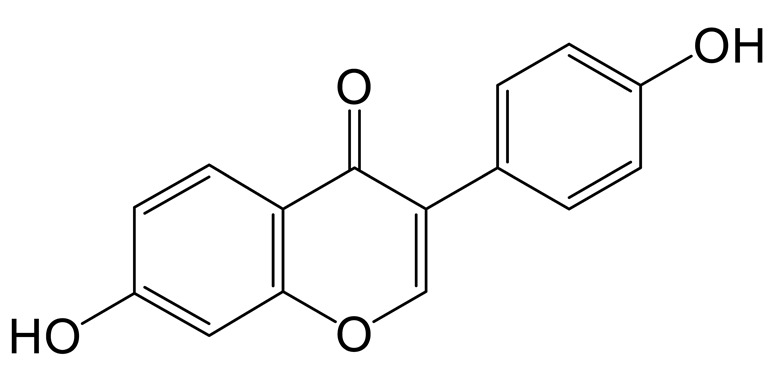	+	−
M1	arabinylation	N	9.39	C_20_H_17_O_9_	401.08777	401.08826	1.134	MS^2^[401]: 121(100), **313(19)**, **225(8)**, **357(2)**, 401(1)	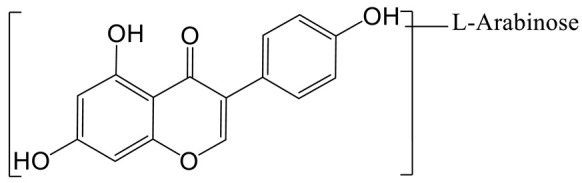	+	+
M2	genistin	N	1.24	C_21_H_19_O_10_	431.09837	431.09836	−0.023	MS^2^[431]: **92(100)**, **162(81**), 120(38), 67(36)	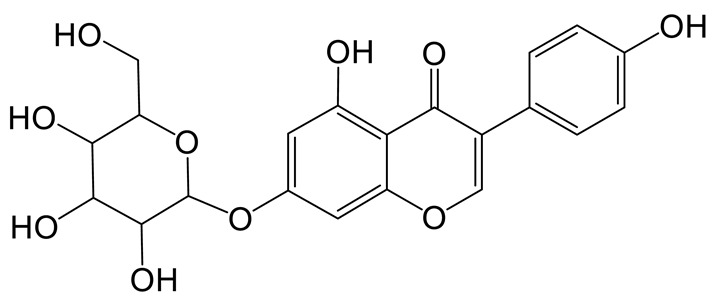	+	−
M3	glucuronidation	N	0.98	C_21_H_17_O_10_	429.08273	429.08463	4.452	MS^2^[429]: **92(100)**, **160(95)**, 197(72), **162(81)**, **187(44)**, 67(36), 56(31)	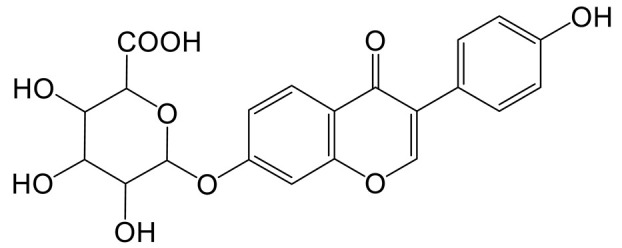	+	−
M4	hydration	N	0.91	C_21_H_21_O_10_	433.11405	433.11371	−0.716	MS^2^[433]: **92(100)**, 160(95), 162(81), 195(75), **197(72)**, 120(38), 67(36), 56(31)	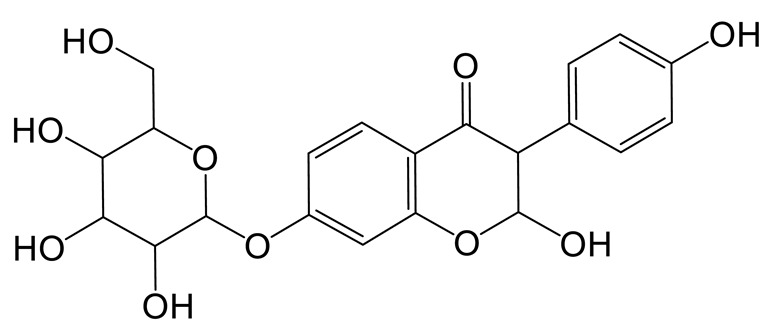	+	−
M5	hydroxylation and methylation	N	9.57	C_22_H_21_O_10_	445.11395	445.11472	1.573	MS^2^[445]: **407(100)**, 408(28), **148(2)**, 104(2)	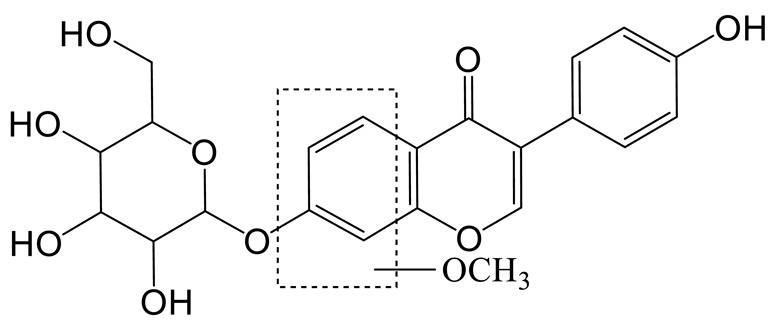	+	−
M6	decarbonylation	P	9.87	C_14_H_11_O_4_	243.06519	243.06551	1.336	MS^2^[243]: **226(100)**, 55(62), **69(53)**, **93(39)**, **121(34)**, **107(29)**, 227(24)	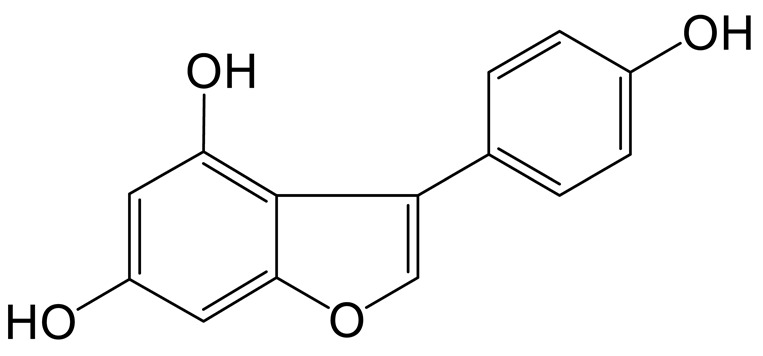	+	+
M7	decarbonylation	P	12.53	C_14_H_15_O_2_	215.10667	215.10724	2.714	MS^2^[215]: 214(100), **146(17**), 55(11),215(11), **149(8)**, 150(3), 56(2)	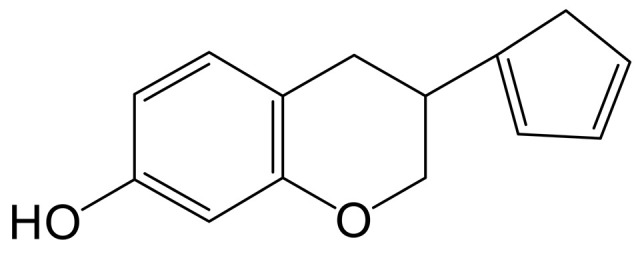	+	−
M8	hydrogenation	P	9.04	C_14_H_13_O_4_	245.08077	245.08086	0.100	MS^2^[245]: **226(100)**, **83(67)**, 81(63), 55(62), **69(53)**, **93(39)**	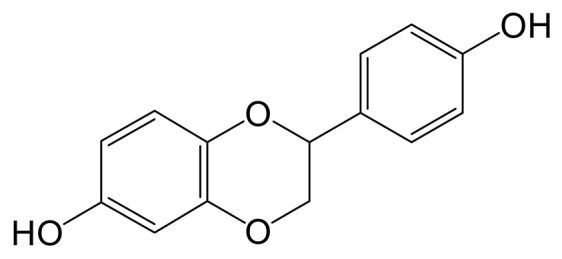	+	−
M9	hydroxylation and methylation	N	10.09	C_22_H_21_O_11_	461.10887	461.10999	2.289	MS^2^[461]: **407(100)**, 408(29), **92(7)**, **425(7)**, 140(3), **137(2)**, 67(2), **84(2)**, 56(1)	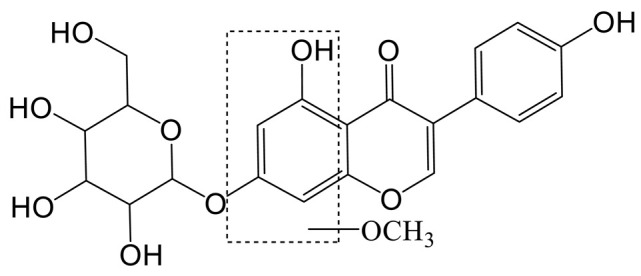	+	+
M10	dehydroxymethylation	N	9.97	C_14_H_7_O_3_	223.04003	223.03984	−1.02	MS^2^[223]: **183(100)**, 184(16), 180(8), 61(5), **111(1)**, **92(1)**	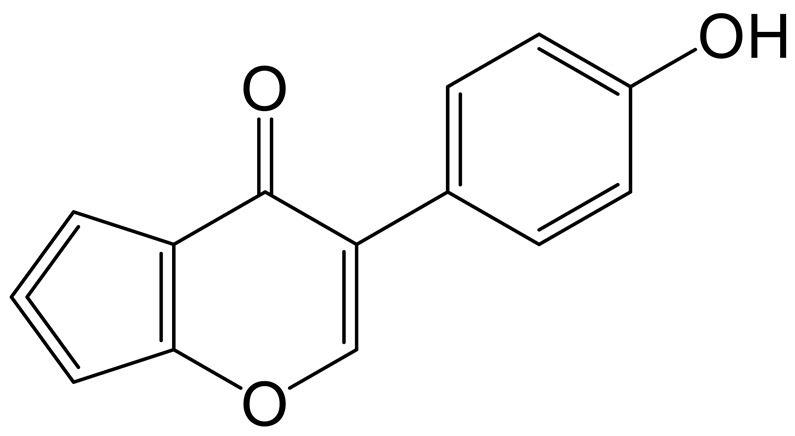	−	+
M11	carbonyl hydrogenation	P	9.37	C_15_H_13_O_4_	257.08088	257.08127	1.691	MS^2^[257]: **69(100)**, 55(75), **189(30)**, 257(22), 107(18)	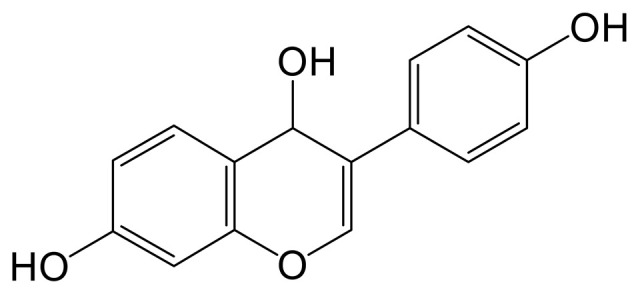	−	+
M12	decarbonylation	N	9.77	C_14_H_9_O_3_	225.05573	225.05559	−0.566	MS^2^[225]: **183(100)**, 184(16), **165(14)**, 92(1)	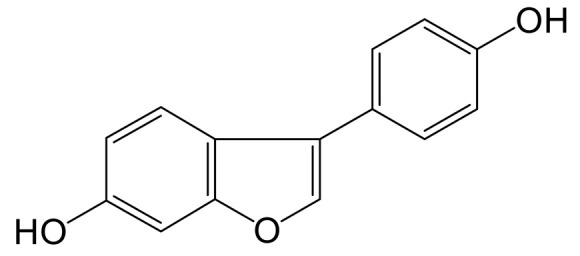	−	+

P: positive ion mode; N: negative ion mode; Con: normal administration group; Mod: hyperlipidemia administration group; +: found in the group; −: not found in the group.

**Table 2:** Identified potential biomarkers regulated by genistin.

Proposed Identity	Fit (%)	Theoretical (*m/z*)	Experimental (*m/z*)	Error (ppm)	Formula	Rt (min)	Ion Mode	Change Trend (Mod/Con)	Change Trend (Gen/Mod)
L-Tryptophan	0.99	205.09619	205.09621	−4.604	C_11_H_12_N_2_O_2_	1.864	[M+H]+	↑ *	↓ ^##^
L-Proline	1.00	116.0703	116.07033	−2.37	C_5_H_9_NO_2_	2.566	[M+H]+	↑ *	↓ ^###^
Octadecanoic acid	0.92	283.26401	283.26425	−0.013	C_18_H_36_O_2_	4.756	[M-H]-	↑ ***	↓ ^###^
4-Amino-4-cyanobutanoic acid	0.93	129.06543	129.06543	−3.208	C_5_H_8_N_2_O_2_	5.565	[M+H]+	↑ **	↓ ^##^
sn-Glycero-3-phosphoethanolamine	0.89	214.04765	214.04778	−3.816	C_5_H_14_NO_6_P	6.486	[M-H]-	↑ **	↓ ^##^
5-Aminolevulinate	1.00	132.06522	132.06537	−1.134	C_5_H_9_NO_3_	5.86	[M+H]+	↑ **	↓ ^###^
L-Leucine	0.99	132.10135	132.10144	−3.522	C_6_H_13_NO_2_	2.416	[M+H]+	↓ ***	↑ ^#^
Creatine	0.99	132.07634	132.07631	−3.128	C_4_H_9_N_3_O_2_	5.395	[M+H]+	↓ ****	↑ ^##^
L-Carnitine	0.91	162.11163	162.11171	−4.687	C_7_H_15_NO_3_	5.798	[M+H]+	↓ **	↑ ^#^
O-Ureido-L-serine	0.87	164.06592	164.06596	−3.793	C_4_H_9_N_3_O_4_	6.203	[M+H]+	↓ ***	↑ ^#^
anserine	0.97	239.1144	239.11438	−2.441	C_10_H_16_N_4_O_3_	6.204	[M-H]-	↓ ****	↑ ^##^
N-Formimino-L-aspartate	0.95	161.05579	161.05566	−0.144	C_5_H_8_N_2_O_4_	6.356	[M+H]+	↓ **	↑ ^##^
L-Ornithine	1.00	133.09659	133.09665	−3.788	C_5_H_12_N_2_O_2_	6.514	[M+H]+	↓ **	↑ ^#^
5-Hydroxyisourate	0.99	183.01495	183.01508	−4.906	C_5_H_4_N_4_O_4_	1.98	[M-H]-	↓ **	↑ ^##^
2,3-Dihydroxycarbamazepine	0.87	269.0932	269.0932	4.204	C_15_H_12_N_2_O_3_	6.336	[M+H]+	↓ *	↑ ^##^

* p < 0.05, ** *p* < 0.01, *** *p* < 0.001, and **** *p* < 0.0001, Con vs. Mod; ^#^ *p* < 0.05, ^##^ *p* < 0.01, and ^###^ *p* < 0.001, Gen vs. Mod. The structures, molecular weights, and codes of differential metabolites were assigned according to the human metabolome database and KEGG compound database

## Data Availability

Most of the data used during the preparation of the manuscript are included in the Results and Discussion sections. However, for any additional details of the procedures and the original raw files, please contact the corresponding authors.

## Supplementary Materials

The following supporting information can be downloaded at: https://www.mdpi.com/article/10.3390/molecules28052242/s1, Figures S1–S3: the metabolic profiles of daidzin, daidzein, genistein; Table S1: approximate values of fold changes of metabolites in the studied groups; Table S2: identified potential biomarkers regulated by genistin and their fragment ion information.

## Sample Availability

Samples of the related compounds are available from the authors.
