# Supplementary material for: The Metabolites and Mechanism Analysis of Genistin against Hyperlipidemia via the UHPLC-Q-Exactive Orbitrap Mass Spectrometer and Metabolomics
Source: Molecules. 2023 Feb 28;28(5):2242. doi: 10.3390/molecules28052242 (PMC10005657; doi:10.3390/molecules28052242)
Supplement: Supplementary file 1 [file molecules-28-02242-s001.zip › supplementary Table S1.pdf]

Supplementary Table 1. Approximate values of fold changes of metabolites in the studied groups.

Proposed identity	Change Trend (Mod/Con)	Change multiples (Mod/Con)	Change Trend (Gen/Mod)	Change multiples (Gen/Mod)
L-Tryptophan	↑*	1.58 times than that in Con group	↓$^{\#\#}$	0.51 times than that in Mod group
L-Proline	↑*	1.73 times than that in Con group	↓$^{\#\#\#}$	0.28 times than that in Mod group
Octadecanoic acid	↑***	2.27 times than that in Con group	↓$^{\#\#\#}$	0.59 times than that in Mod group
4-Amino-4-cyanobutanoic acid	↑**	2.97 times than that in Con group	↓$^{\#\#}$	0.30 times than that in Mod group
sn-Glycero-3-phosphoethanolamine	↑**	2.16 times than that in Con group	↓$^{\#\#}$	0.45 times than that in Mod group
5-Aminolevulinate	↑**	1.86 times than that in Con group	↓$^{\#\#\#}$	0.41 times than that in Mod group
L-Leucine	↓***	0.62 times than that in Con group	↑$^{\#}$	1.32 times than that in Mod group
Creatine	↓****	0.64 times than that in Con group	↑$^{\#\#}$	1.28 times than that in Mod group
L-Carnitine	↓**	0.60 times than that in Con group	↑$^{\#}$	1.64 times than that in Mod group
O-Ureido-L-serine	↓***	0.36 times than that in Con group	↑$^{\#}$	2.03 times than that in Mod group
anserine	↓****	0.15 times than that in Con group	↑$^{\#\#}$	3.80 times than that in Mod group
N-Formimino-L-aspartate	↓**	0.56 times than that in Con group	↑$^{\#\#}$	1.96 times than that in Mod group
L-Ornithine	↓**	0.56 times than that in Con group	↑$^{\#}$	1.62 times than that in Mod group
5-Hydroxyisourate	↓**	0.33 times than that in Con group	↑$^{\#\#}$	1.90 times than that in Mod group
2,3-Dihydroxycarbamazepine	↓*	0.61 times than that in Con group	↑$^{\#\#}$	2.12 times than that in Mod group

$^{*}p < 0.05$, $^{**}p < 0.01$, $^{***}p < 0.001$, and $^{****}p < 0.0001$, Con vs Mod; $^{\#}p < 0.05$, $^{\#\#}p < 0.01$, and $^{\#\#\#}p < 0.001$, Gen vs Mod. Con: control group. Mod: model group. Gen: genistin high dose treatment group.
