# Supplementary material for: The Metabolites and Mechanism Analysis of Genistin against Hyperlipidemia via the UHPLC-Q-Exactive Orbitrap Mass Spectrometer and Metabolomics
Source: Molecules. 2023 Feb 28;28(5):2242. doi: 10.3390/molecules28052242 (PMC10005657; doi:10.3390/molecules28052242)
Supplement: Supplementary file 1 [file molecules-28-02242-s001.zip › supplementary Table S2.pdf]

Supplementary Table 2. Identified potential biomarkers regulated by genistin and their fragment ion information.

Proposed identity	Theoretical (m/z)	Experimental (m/z)	Error (ppm)	Formula	Rt (min)	Ion Mode	MS/MS fragment ions
L-Tryptophan	205.09619	205.09621	-4.604	$C_{11}H_{12}N_2O_2$	1.864	$[M+H]^+$	204, 187, 143, 142, 118
L-Proline	116.0703	116.07033	-2.37	$C_5H_9NO_2$	2.566	$[M+H]^+$	70, 71, 74
Octadecanoic acid	283.26401	283.26425	-0.013	$C_{18}H_{36}O_2$	4.756	$[M-H]^-$	265, 182, 71
4-Amino-4-cyanobutanoic acid	129.06543	129.06543	-3.208	$C_5H_8N_2O_2$	5.565	$[M+H]^+$	83, 56, 112, 84
sn-Glycero-3-phosphoethanolamine	214.04765	214.04778	-3.816	$C_5H_{14}NO_6P$	6.486	$[M-H]^-$	195, 140, 78, 152, 154
5-Aminolevulinate	132.06522	132.06537	-1.134	$C_5H_9NO_3$	5.86	$[M+H]^+$	87, 114, 103, 70, 86
L-Leucine	132.10135	132.10144	-3.522	$C_6H_{13}NO_2$	2.416	$[M+H]^+$	86, 69, 116, 87
Creatine	132.07634	132.07631	-3.128	$C_4H_9N_3O_2$	5.395	$[M+H]^+$	90, 87, 114
L-Carnitine	162.11163	162.11171	-4.687	$C_7H_{15}NO_3$	5.798	$[M+H]^+$	103, 102, 98, 60, 85
O-Ureido-L-serine	164.06592	164.06596	-3.793	$C_4H_9N_3O_4$	6.203	$[M+H]^+$	146, 118, 90, 105, 60
anserine	239.1144	239.11438	-2.441	$C_{10}H_{16}N_4O_3$	6.204	$[M-H]^-$	168, 166, 87, 151, 195, 178
N-Formimino-L-aspartate	161.05579	161.05566	-0.144	$C_5H_8N_2O_4$	6.356	$[M+H]^+$	115, 116
L-Ornithine	133.09659	133.09665	-3.788	$C_5H_{12}N_2O_2$	6.514	$[M+H]^+$	88, 115, 70, 116
5-Hydroxyisourate	183.01495	183.01508	-4.906	$C_5H_4N_4O_4$	1.98	$[M-H]^-$	112, 69, 140, 125, 126, 97
2,3-Dihydroxycarbamazepine	269.0932	269.0932	4.204	$C_{15}H_{12}N_2O_3$	6.336	$[M+H]^+$	241, 239, 213

$^{*}p < 0.05$, $^{**}p < 0.01$, $^{***}p < 0.001$, and $^{****}p < 0.0001$, Con vs Mod; $^{\#}p < 0.05$, $^{\#\#}p < 0.01$, and $^{\#\#\#}p < 0.001$, Gen vs Mod.
