# Supplementary figures and images for: The Metabolites and Mechanism Analysis of Genistin against Hyperlipidemia via the UHPLC-Q-Exactive Orbitrap Mass Spectrometer and Metabolomics
Source: Molecules. 2023 Feb 28;28(5):2242. doi: 10.3390/molecules28052242 (PMC10005657; doi:10.3390/molecules28052242)

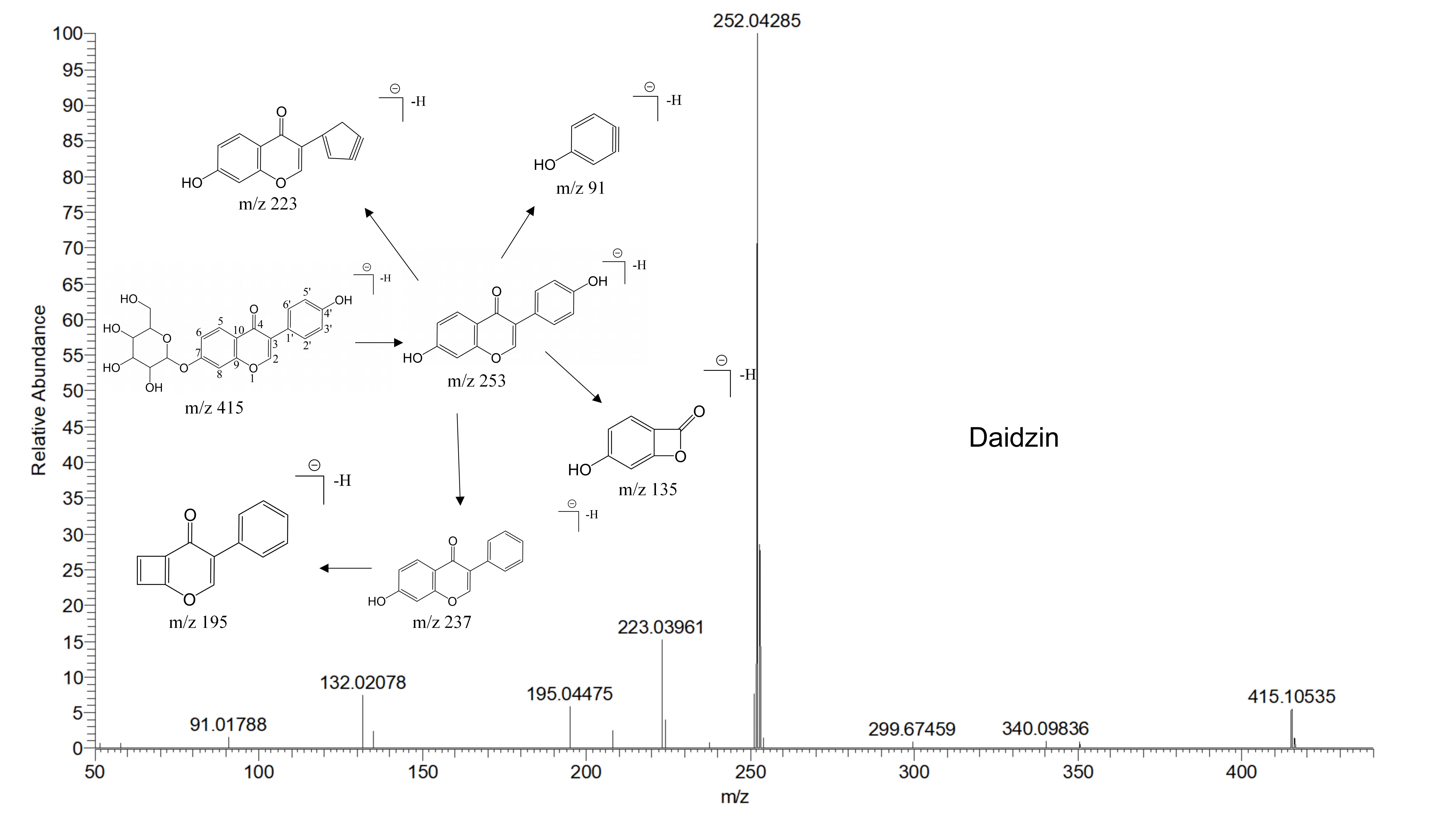

Supplement: Supplementary file 1 [file molecules-28-02242-s001.zip › supplementary Figure S1.jpg]

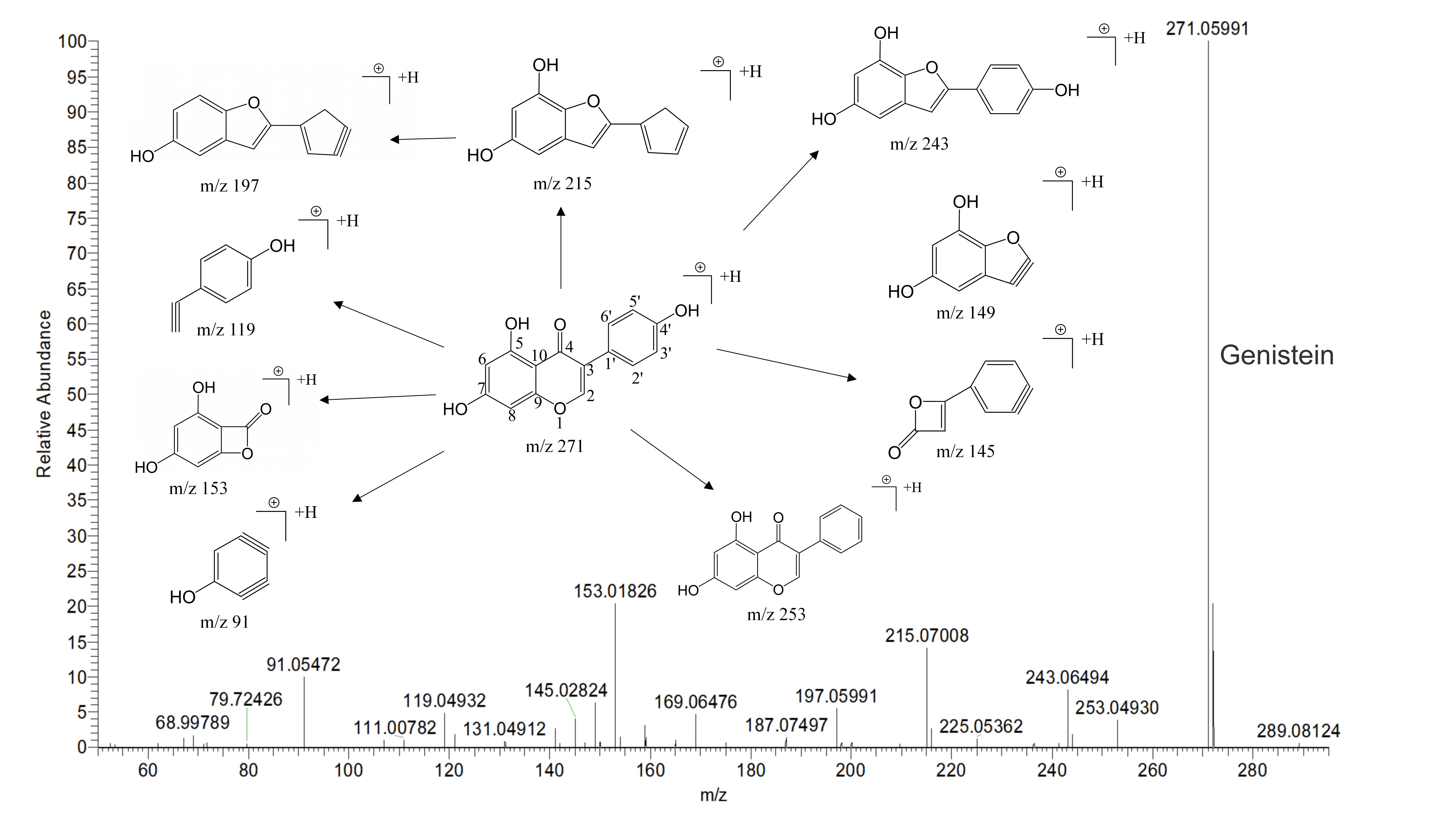

Supplement: Supplementary file 1 [file molecules-28-02242-s001.zip › supplementary Figure S2.jpg]

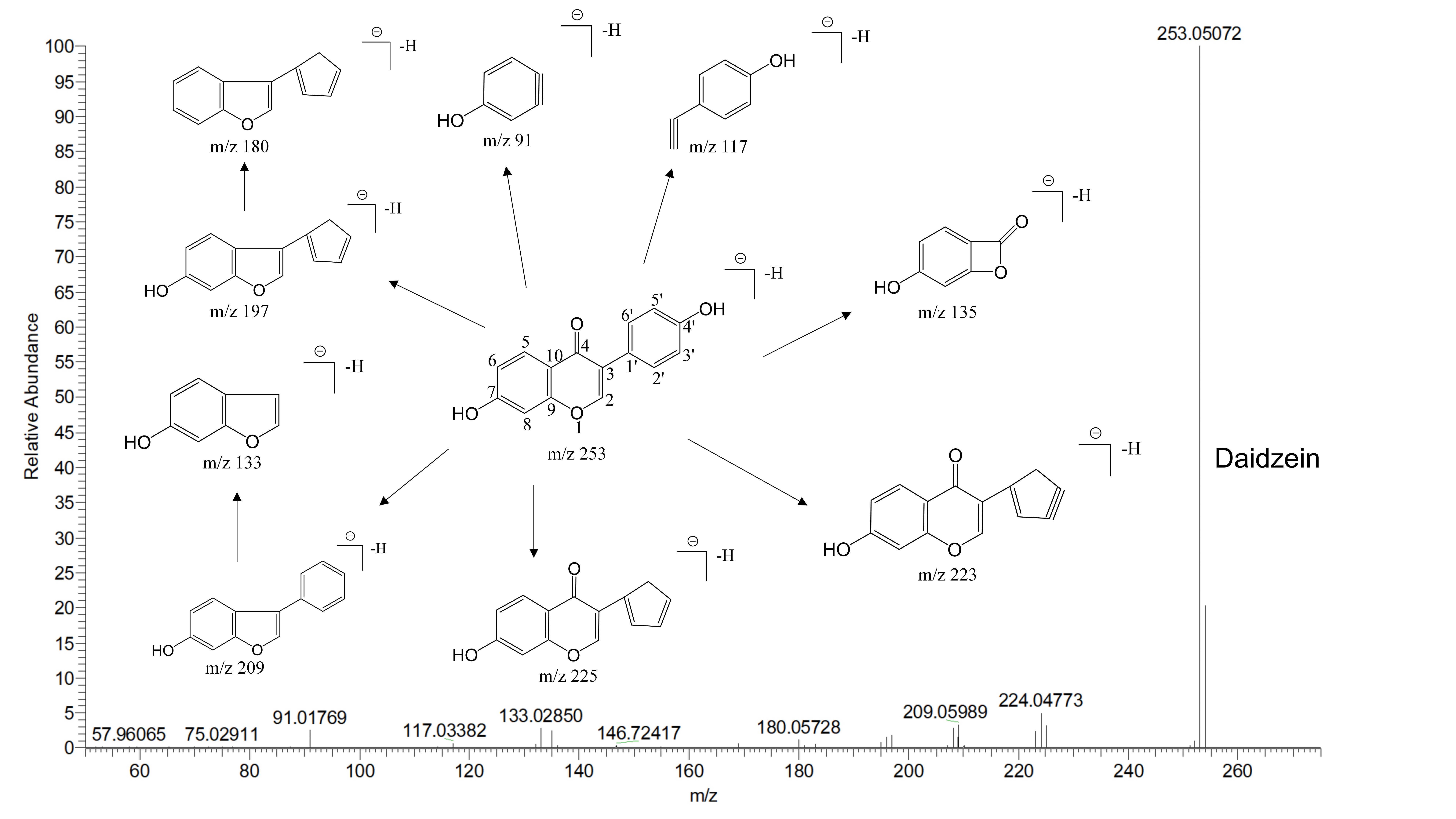

Supplement: Supplementary file 1 [file molecules-28-02242-s001.zip › supplementary Figure S3.jpg]
